# Validation of a Multiplex Allele-Specific Polymerase Chain Reaction Assay for Detection of *KRAS* Gene Mutations in Formalin-Fixed, Paraffin-Embedded Tissues from Colorectal Cancer Patients

**DOI:** 10.1371/journal.pone.0147672

**Published:** 2016-01-26

**Authors:** Sirirat Seekhuntod, Paninee Thavarungkul, Nuntaree Chaichanawongsaroj

**Affiliations:** 1 Department of Transfusion Medicine and Clinical Microbiology, Faculty of Allied Health Sciences, Chulalongkorn University, Bangkok, Thailand; 2 Department of Medical Services, Institute of Pathology, Ministry of Public Health, Bangkok, Thailand; Sapporo Medical University, JAPAN

## Abstract

**Background:**

Patients with *KRAS* mutations do not respond to epidermal growth factor receptor (EGFR) inhibitors and fail to benefit from adjuvant chemotherapy. Mutation analysis of *KRAS* is needed before starting treatment with monoclonal anti-EGFR antibodies in patients with metastatic colorectal cancer (mCRC). The objective of this study is to develop a multiplex allele-specific PCR (MAS-PCR) assay to detect *KRAS* mutations.

**Methods:**

We developed a single-tube MAS-PCR assay for the detection of seven *KRAS* mutations (G12D, G12A, G12R, G12C, G12S, G12V, and G13D). We performed MAS-PCR assay analysis for *KRAS* on DNA isolated from 270 formalin-fixed paraffin-embedded (FFPE) colorectal cancer tissues. Sequences of all 270 samples were determined by pyrosequencing. Seven known point-mutation DNA samples diluted with wild-type DNA were assayed to determine the limitation of detection and reproducibility of the MAS-PCR assay.

**Results:**

Overall, the results of MAS-PCR assay were in good concordance with pyrosequencing, and only seven discordant samples were found. The MAS-PCR assay reproducibly detected 1 to 2% mutant alleles. The most common mutations were G13D in codon 13 (49.17%), G12D (25.83%) and G12V (12.50%) in codon 12.

**Conclusion:**

The MAS-PCR assay provides a rapid, cost-effective, and reliable diagnostic tool for accurate detection of *KRAS* mutations in routine FFPE colorectal cancer tissues.

## Introduction

Colorectal cancer (CRC) is the most common cancer and the third leading cause of cancer death in the world [1]. In Thailand, CRC is the third most common cancer among men and the fifth most common one among women [2, 3]. One of the major molecular pathways in CRC development is the induction of an activating mutation in the proto-oncogene *KRAS* (Kirsten rat sarcoma viral oncogene) [4, 5]. The *KRAS* gene is a member of the *RAS* gene family and encodes a 21-kDa RAS protein, which is a downstream GTP-binding protein in the epidermal growth factor receptor (EGFR) signal transduction pathway. The oncogenic forms of *KRAS* mutations constitutively express the active RAS protein leading to increased cell division, cell proliferation, prevention of apoptosis process, induction of angiogenesis and increased metastasis [6]. Recently, cancer therapies have been developed using monoclonal antibodies, including cetuximab and panitumumab, to target the EGFR [7, 8]. These agents are designed to block ligand-induced EGFR tyrosine kinase activation and, thus, inhibit downstream signaling [9]. However, only the CRC with wild-type *KRAS* proto-oncogene responds to anti-EGFR antibodies treatment, whereas no therapeutic response occurs in CRC with *KRAS* mutations [9–11]. The European Society for Medical Oncology and the American Society of Clinical Oncology have established major oncology guidelines that these antibodies be restricted to patients with *KRAS* wild-type colorectal cancers [12, 13]. Therefore, detection of *KRAS* gene mutations has critical clinical relevance for developing individualized patient therapeutic strategies [7].

Several molecular methods have been developed for detecting *KRAS* mutations. These methods include direct sequencing [10], real-time PCR [11], high resolution melting (HRM) [14], amplification refractory mutation system polymerase chain reaction [15, 16], pyrosequencing [17, 18], co-amplification at lower denaturation temperature PCR [19], mutant-enriched PCR [20], and digital PCR [21]. In addition, several commercial molecular kits are commonly available for *KRAS* mutation detection, including the cobas^®^ KRAS Mutation Test [22], 3D-Gene^®^ KRAS mutation assay kit, therascreen^®^ KRAS RGQ PCR Kit [23], EntroGen’s KRAS Mutation Analysis Kit for Real-Time PCR [23], and KRAS PyroMark Q96 V2.0 Kit [24]. However, all of these methods require technical expertise and specialized equipment and instruments, and are too expensive as prognostic and diagnostic tools for cancer patients in developing countries. By contrast, Multiplex allele-specific Polymerase Chain Reaction (MAS-PCR) is a simple, reliable and inexpensive method for detection of known mutations and single-nucleotide polymorphism [25, 26]. MAS-PCR is characterized by primers with an allele-specific 3’ terminus that anneals specifically to mutated or wild-type DNA template only [26, 27]. Wild-type and allele-specific primers generate different sized PCR products permitting easy detection of a known gene mutation.

In this study, we developed a MAS-PCR assay for analysis of the mutational status of *KRAS* codons 12 and 13. Single nucleotide point mutations in the *KRAS* gene occur most frequently in codons 12 and 13 accounting for 80 to 82% and 15 to 17% of the mutations, respectively [28–31]. Mutations in other positions, such as codons 61, 117, 146 and 154, are much less frequent amounting to approximately 1% of all *KRAS* gene mutations [17, 32]. In this study, presence of the most common point mutations in codons 12 and 13, which are G12D, G12A, G12R, G12C, G12S, G12V, and G13D [18, 30, 32, 33], was assessed in formalin-fixed, paraffin-embedded tissue samples from 270 CRC patients. Pyrosequencing, a robust and sensitive method, was used as a reference method to compare the sensitivity of MAS-PCR assay for detection of the *KRAS* mutant alleles.

## Materials and Methods

### Preparation of Clinical Samples

Formalin-fixed, paraffin-embedded colorectal adenocarcinomas from 270 patients with CRC were collected from the Institute of Pathology, Ministry of Public Health, Bangkok, Thailand. The study was approved by the Ethics Committee of the Institute of Pathology (IOP-KM-R57-007). The ethics committee waived the need for consent because the data of tissue samples were analyzed anonymously and reported. An experienced pathologist reviewed and marked the adenocarcinoma areas of the hematoxylin and eosin stained slides. Tumors were manually micro-dissected from paraffin-embedded blocks, and 10 μm thick sections were collected in a 1.5 ml tube. Paraffin was removed from the tissue blocks with xylene, and samples were air-dried. DNA was extracted from the samples and purified using a QIAamp DNA FFPE Tissue Kit (QIAGEN, Hilden, Germany) according to the manufacturer’s instructions. DNA quantity was determined by NanoDrop spectrophotometry (NanoDrop Technologies, Wilmington, DE).

### PCR Amplification and Pyrosequencing

Pyrosequencing for analysis of a *KRAS* gene fragment spanning codons 12 and 13 was performed as previously described with some modifications [18]. Sequences of the primers were previously described [18]. Reaction conditions with 1 μM forward primer (5’-GGCCTGCTGAAAATGACTGAA-3’), biotinylated reverse primer (5’-biotin-TTAGCTGTATCGTCAAGGCACTCT-3’), and 50 to 100 ng/μl DNA template yielded a 82-bp product. Reactions were performed in 1x PCR buffer containing 2.5 mM MgCl_2_, 0.2 mM dNTP (BioLabs, England), and 0.625 U Amplitag Gold DNA Polymerase (Applied Biosystems, USA) in a 30 μl total volume. PCR conditions were a denaturing step at 95°C for 10 min, then 1 min at 95°C, 1 min at 55°C, 1 min at 72°C for 35 cycles, and followed by 7 min at 72°C. PCR products were confirmed by 8% polyacrylamide gel electrophoresis at 140 V for 40 min, and gels were stained with SYBR Green I Nucleic Acid Gel Stain (1:400, Lonza, USA) for 30 min. The PCR products were sequenced by pyrosequencing using the PyroMark Gold Q96 reagent (Qiagen, Germany). PCR products in 30 μl were mixed with 3 μl streptavidin-conjugated Sepharose beads (Streptavidin Sepharose HP, Amersham Biosciences AB, Sweden), 40 μl binding buffer and 17 μl distilled water, followed by shaking at 1400 rpm for 10 min. The immobilized biotinylated PCR products: streptavidin-conjugated Sepharose beads complexes were captured using a vacuum prep tool. Single-stranded DNA purification was achieved by 1x washing the vacuum prep tool sequentially with 70% ethanol for 5 s, denaturation solution for 5 s, and washing buffer for 10 s. Biotinylated single-stranded DNA was added to a 96-well microtiter plate that contained 40 μl of 0.4 μM sequencing PF1-primer (5’-TGTGGTAGTTGG AGCTG-3’) for analysis at nucleotides 35 and 38 positions, and PF2-primer (5’-TGTGGTAGTTGGAGCT-3’) for analysis at nucleotide 34 position [18]. The plate was incubated at 80°C for 2 min, followed by cooling to room temperature for 5 min, and loaded onto the PyroMark Q96 ID system (Qiagen, Germany).

### DNA Cloning

Genomic DNA of eight clinical samples harboring *KRAS* wild-type DNA and seven point mutations in *KRAS* codons 12 and 13 (G12D, G12A, G12R, G12C, G12S, G12V, and G13D) were subjected to PCR amplification using universal *KRAS* primers (K-*ras*-codon 12/13-F 5’-CTG GTG GAG TAT TTG ATA GTG TAT T-3’ and K-*ras*-codon 12/13-R 5’-ATC TGT ATC AAA GAA TGG TCC TG-3’). All 259-bp PCR products were cloned into psc-A-amp/kan vector and transformed into competent *Escherichia coli* cells using a Strataclone PCR cloning kit (Agilent Technologies; United States). The transformed bacteria were spread onto selective LB-agar plates (Oxoid, USA) with ampicillin and X-Gal (Promega, USA). After an overnight incubation at 37°C, white colonies were selected randomly and cultured in LB medium, overnight. Plasmids were extracted using Wizard^®^ genomic DNA purification kit (Geneaid, Taipei, Taiwan) and subsequently screened for the insert fragment by PCR. Positive PCR products were sequenced by the Bioneer Corporation, Daejeon, Republic of Korea.

### Primer Design

**Fig 1:**
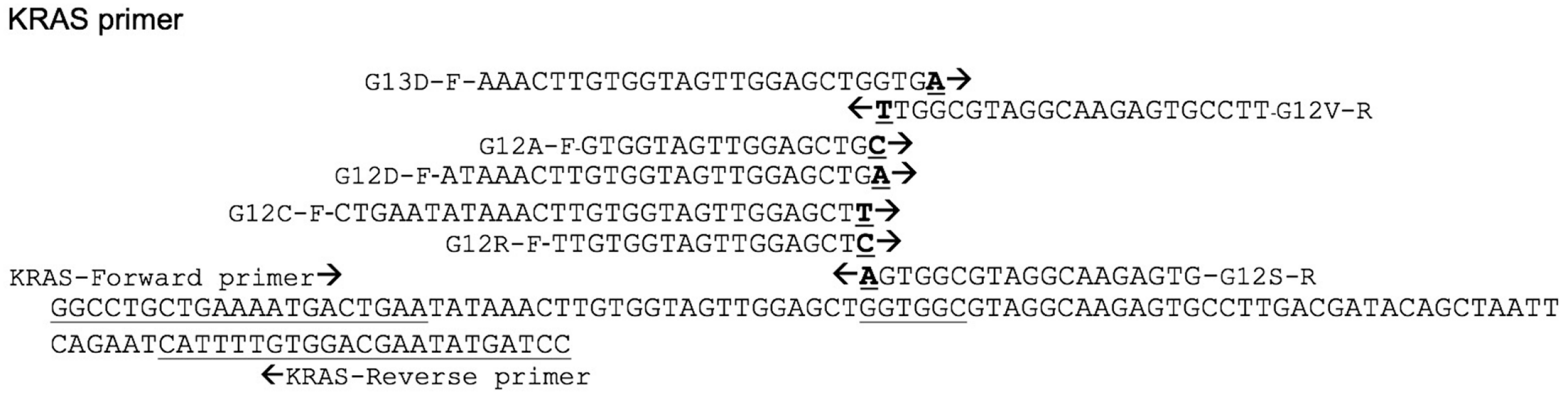
Target region of *KRAS* gene at codons 12 and 13 for amplification and the design of primers. The positions of primers are illustrated. AS primers share the same forward primer or reverse primer. Solid arrows indicate the forward and reverse primers. The 3′ terminal base of each AS primers was adapted according to its corresponding mutation, and is bold and underlined. Codons and mutated bases are underlined.

Allele-specific (AS) primers were designed for each of seven mutations, and a mutation-unspecific region was used as a reference amplicon. The 3′ terminal base of each AS primer was adopted according to its corresponding mutation. Amplification reactions were performed with the main *KRAS* forward primer and five AS primers (G12R-F, G12C-F, G12D-F, G12A-F, and G13D-F) sharing with one common antisense *KRAS* reverse primer, and reactions with two AS-primers (G12S-R and G12V-R) sharing with one common sense *KRAS* forward primer (Fig 1). Sequences of the primers used in this study are listed in Table 1. All primers were synthesized and supplied by BioDesign Co., Ltd. (BioDesign, Pathumthani, Thailand).

**Table 1:** Primers used in MAS-PCR for detecting the most common mutations in codons 12 and 13 of *KRAS* gene.

Primer	Sequence	Conc. (μM)	Product length (bp)
*KRAS*-F	5’-GGCCTGCTGAAAATGACTGAA-3’	0.05	113 bp
*KRAS*-R	5’-GGATCATATTCGTCCACAAAATG-3’	0.075	113 bp
G12S-R	5’-CACTCTTGCCTACGCCACT-3’	0.05	64 bp
G12R-F	5’-TTGTGGTAGTTGGAGCTC-3’	0.025	85 bp
G12C-F	5’-CTGAATATAAACTTGTGGTAGTTGGAGCTT-3’	0.01	97 bp
G12D-F	5’- ATAAACTTGTGGTAGTTGGAGCTGA-3’	0.02	91 bp
G12A-F	5’- GTGGTAGTTGGAGCTGC-3’	0.02	83 bp
G12V-R	5’- AAGGCACTCTTGCCTACGCCAA-3’	0.02	68 bp
G13D-F	5’- AAACTTGTGGTAGTTGGAGCTGGTGA-3’	0.0125	89 bp

### Multiplex Allele-Specific PCR (MAS-PCR) Assay

**Fig 2:**
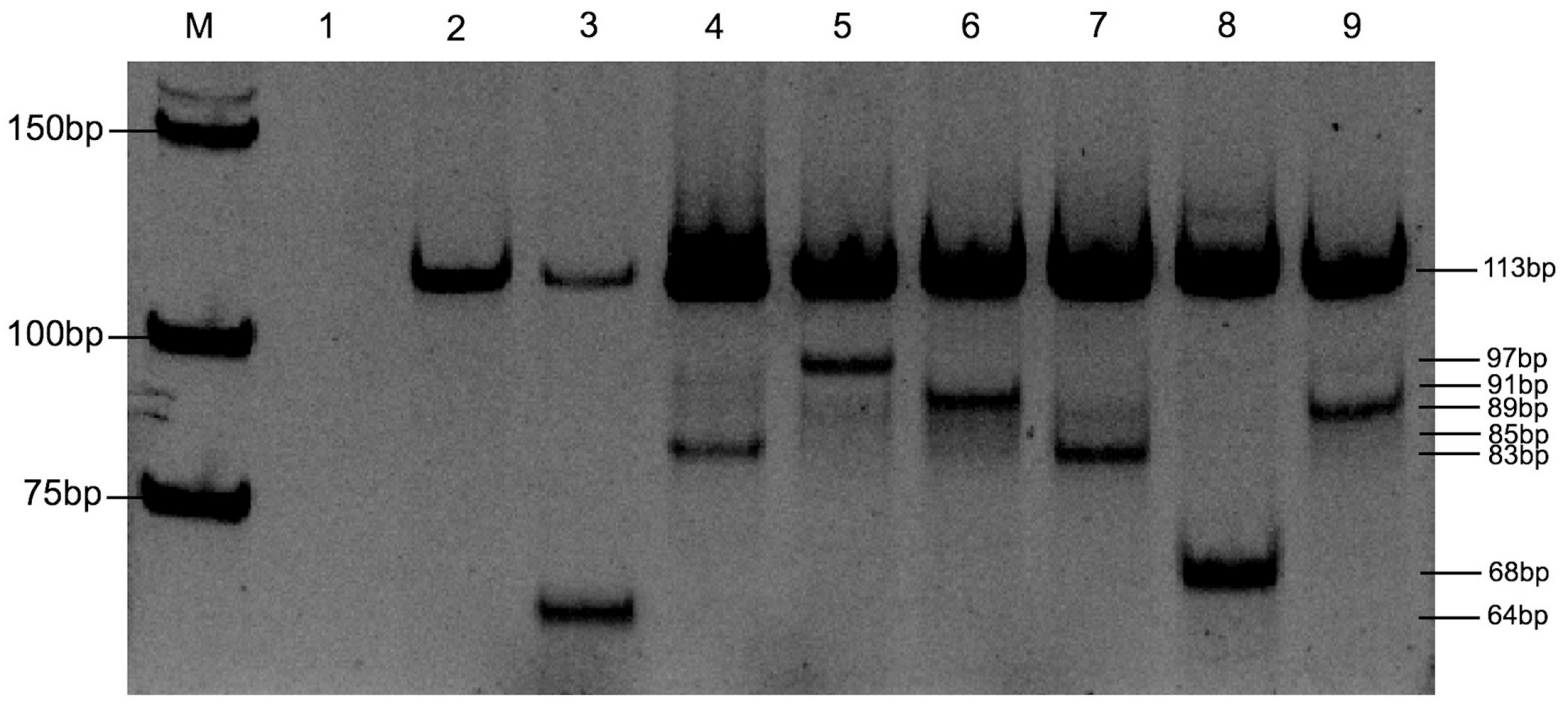
MAS-PCR assay of *KRAS* gene. Assay distinguished wild-type *KRAS* gene codons 12 and 13 from different mutants using AS primers. Lane M: Low molecular weight DNA ladder; Lane 1: negative control; Lane 2: wild-type; Lane 3: G12S mutant; Lane 4: G12R mutant; Lane 5: G12C mutant; Lane 6: G12D mutant; Lane 7: G12A mutant; Lane 8: G12V mutant; Lane 9: G13D mutant.

A single reaction had two main *KRAS* primers and seven AS primers targeting on seven mutated nucleotides within the *KRAS* gene. The MAS-PCR reaction in 50 μl total volume contained 1x PCR buffer, 2.5 mM MgCl_2_, 0.2 mM dNTP (BioLabs, England), 0.625 U Amplitag Gold DNA Polymerase (Applied Biosystems, USA), 50 to 100 ng/μl DNA template. The optimized concentrations of each primer are shown in Table 1. The reaction was amplified under the following conditions: an initial denaturation step at 95°C for 10 min, followed by 10 cycles at 95°C for 30 s, 60°C for 45 s, 72°C for 60 s, a second step of 20 cycles at 95°C for 30 s, 64°C for 45 s, 72°C for 60 s, and a third step of 20 cycles at 95°C for 30 s, 55°C for 45 s, 72°C for 60 s, and finally 10 min at 72°C. After amplification, the amplicons with a loading volume of 20 μl were analyzed by 8% polyacrylamide gel electrophoresis at 140 V for 60 min, and gels were stained with SYBR Green I Nucleic Acid Gel Stain (1:400, Lonza, USA) for 30 min. Because of the 3′ end of each AS primer pairs with the respective nucleotide base in the mutant sequence of *KRAS* gene, the allele-specific fragment was amplified yielding one of 97-bp, 91-bp, 89-bp, 85-bp, 83-bp, 68-bp and 64-bp products (represented the G12C, G12D, G13D, G12R, G12A, G12V, and G12S mutants, respectively) along with the positive 113-bp band as an internal control. While wild-type DNA at any of the seven positions prevents allele-specific amplification resulting in a corresponding missing band (Fig 2).

### Sensitivity of MAS-PCR Assay

Eight plasmid clones of *KRAS* wild-type and seven *KRAS* mutants (G12D, G12A, G12R, G12C, G12S, G12V, or G13D) were extracted. Each mutated plasmid DNA was mixed with a wild-type plasmid DNA in total of 100 ng. The proportion of mutant plasmid DNA was gradually reduced to obtain decreasing ratios of mutant to wild-type DNA at 100%, 50%, 25%, 10%, 5%, 2%, 1% and 0.1%. The precision and reproducibility were determined in four repeated run by analyzing the mixtures at the range of lowest detection limit which were demonstrated by MAS-PCR.

### Data analysis

Results obtained from MAS-PCR and pyrosequencing were compared for the 270 formalin-fixed, paraffin-embedded specimens and were evaluated for significance using the Kappa statistics. A value of k > 0.81 was considered to be significant and to indicate that both methods provide almost perfect results. Positive and negative agreement confident intervals were determined for the MAS-PCR results. Differences in the categorical variables such as age, gender, histologic grade and site of tumor between patients and with *KRAS* mutations were evaluated for significance with chi-square test. Statistical tests were two-sided, and p < 0.05 was considered significant. Statistics were carried out using SPSS software (version 11.5).

## Results

### Pyrosequencing analysis of *KRAS* gene mutations in CRC specimens

Two hundred and seventy clinical samples were first analyzed by pyrosequencing for 6 different point mutations in codon 12 (G12S, G12R, G12C, G12D, G12A, and G12V) and one point mutation in codon 13 (G13D) of the *KRAS* gene. The mutations selected for this study are among the most frequently found in colorectal cancer patients. Of the 270 tissue specimens, the sequencing results revealed that 120 cases (44.44%) had a mutation in either codon 12 or 13 in the *KRAS* gene, while 150 cases (55.55%) were defined as *KRAS* wild type (Table 2). Of the 120 cases with a *KRAS* mutation, 61 (50.83%) patients had a codon 12 mutation. The most frequent codon 12 mutation was G12D at 25.83%, followed by G12V at 12.50%. The incidence of G12A, G12S and G12C mutations ranged from 6 to 3%, while that of G12R was the lowest at < 1%. The codon 13 mutation G13D found at 49.17% was more prevalent than any of the codon 12 mutations. Patients had either one or no detectable mutation.

**Table 2:** *KRAS* mutational status in 270 CRC patients.

*KRAS* status	Type of mutations Amino acid change	Pyrosequencing Number of mutations (%)	MAS-PCR Number of mutations (%)
Mutant	All Codon 12 and 13	120/270	113/270
		(44.44%)	(41.85%)
Mutated Codon 12	All Codon 12	61/120	54/113
		(50.83%)	(47.79%)
G12D	GGT>GAT	31/120	28/113
	Gly→Arg	(25.83%)	(24.78%)
G12V	GGT>GTA	15/120	15/113
	Gly→Val	(12.50%)	(13.27%)
G12A	GGT>GCT	7/120	4/113
	Gly→Ala	(5.83%)	(3.54%)
G12S	GGT>AGT	3/120	3/113
	Gly→Ser	(2.50%)	(2.65%)
G12C	GGT>TGT	4/120	4/113
	Gly→Cys	(3.33%)	(3.54%)
G12R	GGT>CGT	1/120	0/113
	Gly→Arg	(0.83%)	ND
G13D	GGC>GAC	59/120	59/113
	Gly→Asp	(49.17%)	(52.21%)

Underlined bases represent the substitutions in the respective codon.

ND denotes not detected.

### MAS-PCR analysis of *KRAS* gene mutations in CRC clinical samples

The MAS-PCR assay, performed on the 270 CRC tissue specimens to detect the *KRAS* codon 12 and codon 13 mutations, revealed results that closely agreed with those of pyrosequencing. As shown on Table 2, 113 (41.85%) cases of *KRAS* mutation in codons 12 and 13 and 157 cases (58.15%) of *KRAS* wild type were identified by MAS-PCR assay. Among the 113 mutated cases, 47.79% had a mutation at codon 12. Moreover in reference to pyrosequencing results, seven mutated cases (three G12D, one G12R, and three G12A) were not detected by MAS-PCR assay. However all codon 13 mutations were correctly identified, as with pyrosequencing results.

### Correlation of patients’ characteristics with *KRAS* codon 12 and 13 mutations

The median age of patients was 62 years ranging from 27 to 90 years-old. Majority of the patients were between 60 and 79 years of age (55.19%). Male to female ratio was 1.55:1. The dominant histological type at 64.81% (175/270) was moderately differentiated, and 88.52% of the cases (239/270) were colorectal primary tumors (Table 3). A possible correlation between patients’ demographic characteristics and detected *KRAS* mutations was examined as shown in Table 3. Of the studied patients presenting *KRAS* mutated carcinoma, no significant differences were found with regard to age, gender, histologic grade and tumor site. Mutations in codons 12 and 13 were fairly and evenly distributed within the groups being close to 1:1 ratio regardless of the characteristic.

**Table 3:** Correlation between *KRAS* mutation and Patients’ characteristics of 270 colorectal carcinomas.

Characteristics	N (%)	WT KRAS N (%)	MT KRAS N (%)	P Value	Codon 12 mutated N (%)	Codon 13 mutated N (%)	P Value
**Total patients**	270						
Median age (range)	62						
20–39 years	11 (4.07%)	7 (63.64%)	4 (36.36%)	0.117	1 (25.00%)	3 (75.00%)	0.268
40–59 years	98 (36.30%)	48 (48.98%)	50 (51.02%)		28 (56.00%)	22 (44.00%)	
60–79 years	149 (55.19%)	85 (57.05%)	64 (42.95%)		30 (46.88%)	34 (53.12%)	
80–90 years	12 (4.44%)	10 (83.33%)	2 (16.67%)		2 (100%)	0	
**Gender**:							
Male	164 (60.74%)	95 (57.93%)	69 (42.07%)	0.329	36 (52.17%)	33 (47.83%)	0.733
Female	106 (39.26%)	55 (51.89%)	51 (48.11%)		25 (49.02%)	26 (50.98%)	
**Histologic Grade**:							
Well differentiated	77 (28.52%)	35 (45.45%)	42 (54.55%)	0.057	19 (45.24%)	23 (54.76%)	0.641
Moderate differentiated	175 (64.81%)	102 (58.29%)	73 (41.71%)		39 (53.42%)	34 (46.58%)	
Poorly differentiated	18 (6.67%)	13 (72.22%)	5 (27.78%)		3 (60.00%)	2 (40.00%)	
**Site**:							
Colorectal primary	239 (88.52%)	128 (53.56%)	111 (46.44%)	0.066	55 (49.55%)	56 (50.45%)	0.323
Metastasis	31 (11.48%)	22 (70.97%)	9 (29.03%)		6 (66.67%)	3 (33.33%)	

### Comparison of MAS-PCR and pyrosequencing

Results for the 270 FFPE samples were subjected to agreement analyses, and the MAS-PCR findings were compared to those of the pyrosequencing method (Table 4). Concordant results of 263 out of 270 samples showed that both methods provided perfect results (p < 0.05) with no statistically significant difference between the assays (k = 0.947, 95% CI = 0.909 to 0.986). For 7 cases, discordant results were obtained, mainly regarding codon 12 mutations which were detectable by pyrosequencing and sanger direct sequencing but not with MAS-PCR assay. The positive agreement and negative agreement were 94% and 100%, respectively.

**Table 4:** Pairwise comparison and agreement analyses between MAS-PCR and pyrosequencing.

	Pyroseqencing		
	Number Mutant	Number Wild-type	Totals
**MAS-PCR**			
Number Mutant	113	0	113
Number Wild-type	7	150	157
**Totals**	120	150	270

Positive agreement: 94% Cl 95% [88%; 97%]

Negative agreement: 100% Cl 95% [98%; 100%]

### Sensitivity, precision and reproducibility of the MAS-PCR for *KRAS* mutation

**Fig 3:**
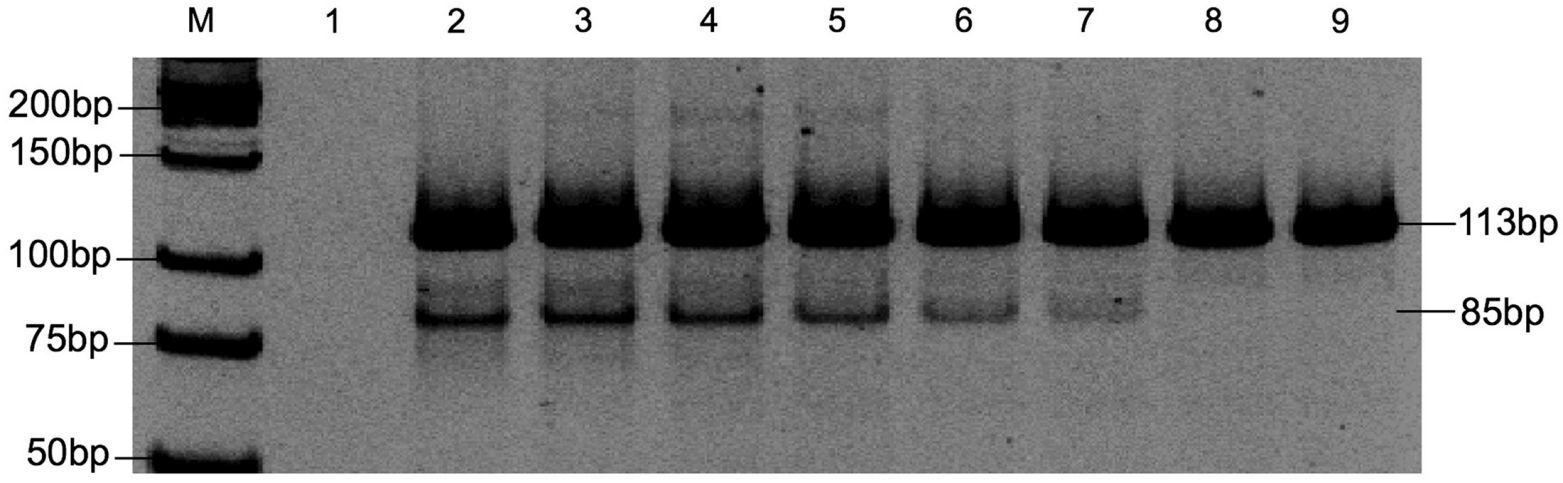
Sensitivity of MAS-PCR assay for identifying *KRAS* gene mutations. A representative gel is shown. Dilutions of G12R mutant plasmid and wild-type plasmid DNA (from 100%, 50%, 25%, 10%, 5%, 2%, 1% and 0.1% mutated alleles) Lane M: Low molecular weight DNA Ladder; Lane 1: negative control; Lane 2–9: corresponded to PCR products from 100% to 0.1%.

To evaluate the sensitivity of the MAS-PCR assay, plasmid DNA of each seven *KRAS* mutant clones was diluted in separate amplification reactions with plasmid DNA of a wild-type *KRAS* clone. The proportion of mutant DNA was gradually reduced to obtain decreasing ratios of mutant to wild-type DNA. The MAS-PCR assay detected mutant alleles down to 1% for the G12S mutant, and 2% for the G12R, G12C, G12D, G12A, G12V, and G13D mutants. The representative example of MAS-PCR assay for lowest limit of detection is shown in Fig 3. To test for precision and reproducibility of our MAS-PCR assay, we quantified *KRAS* mutations in the DNA mixtures containing each of the seven mutant *KRAS* DNA and wild-type DNA at varying ratios (10%, 5%, 2%, 1%, 0.1% and 0.01%) in four repeated runs. The results were precise and reproducible, of which the same lowest amount of *KRAS* mutant alleles were detected in all repeated runs.

## Discussion

Many studies have examined the association of *KRAS* mutations with CRC [8, 33–37]. *KRAS* mutations in CRC patients correlate with resistance to anti-EGFR treatment, such as cetuximab or panitumumab [7]. Hence, accurate prediction of therapeutic responses will spare patients from unnecessary treatment while focusing on more individualized effective therapy. A wide variety of methods have been developed, and several commercial molecular kits are commonly available for detecting *KRAS* mutations [10, 11, 14, 17, 20, 23]. Each technique has its own set of problems and issues. For example, direct sequencing is the most commonly used approach to screen for *KRAS* mutations, however sensitivity for detecting mutant DNA is low and requires at least 10%-30% of mutated alleles in a wild-type background [14, 18, 38]. HRM is a rapid methodology that enables high-throughput screening of *KRAS* mutations with a moderate analytical sensitivity of 5% to 6% [14], however, its main limitation is the inability to identify which codon is mutant. HRM results should be confirmed, and identification of specific mutations requires another technique, such as direct sequencing [39]. Pyrosequencing is accurate, feasible and has superior analytical sensitivity of approximately 5% mutant allele [14, 18]. Nevertheless, this assay requires a costly instrument, and expensive reagents and consumables, making it cost prohibitive for use in developing countries. Commercial molecular kits have various advantages, including high sensitivity (i.e., detection limit around 1% to <5%), speed, easy data interpretation, and numerous detectable positions of *KRAS* mutations, however this assay also requires a costly instrument, expensive reagents, and has a relatively high cost per sample [40]. Therefore, there is a need to develop an accurate, simple, and cost-effective method to detect *KRAS* mutations known to be associated with CRC that can be utilized in developing countries.

In this study, we successfully developed highly sensitive and specific MAS-PCR assay, targeting the seven (i.e., G12S, G12R, G12C, G12D, G12A, G12V, and G13D) most common mutations in codons 12 and 13 of *KRAS* gene. We designed specific primers for each mutation, and a mutation-nonspecific region was used as a reference amplicon. The 3′-terminal base of each AS primer was adopted according to its corresponding mutation. The MAS-PCR assay was optimized for each primer in terms of the primer concentration and amplification parameters. The MAS-PCR assay we reported herein is the first development of multiplex allele-specific PCR employing the standard PCR method that could detect the seven most common *KRAS* gene mutations. The method could simply be performed in low resource laboratories. Further investigation is on the way to develop a simple, rapid and user-friendly Nucleic Acid Lateral Flow (NALF) immunoassay [41] using biotinylated primers based on primers developed in this study. Moreover probe-based real-time PCR could be established using our MAS-PCR primer set to detect *KRAS* mutations. Both methods could be advantageous in terms of preventing crosstalk between samples and/or environmental contaminations during conducting experimentations.

In the present study, we observed that the frequency of *KRAS* oncogene mutations in codons 12 and 13 in 270 samples of colorectal cancer Thai patients was 44.44%. Similar frequencies, ranging from 20 to 50%, have been previously described [28, 30, 33]. We found the rates of mutations in codon 12 and codons 13 were at 50.83% and 49.17%, respectively, which were higher than those reported in other studies (codon 12: 70–90%, codon 13: 10–30%) [28, 30, 31, 33]. However, our results gave similar frequencies (codon 12: 52.62% (10/19), codon 13: 42.12% (8/19)) as reported by Poehlmann et al [17]. The frequently found G13D, G12D, and G12V mutations identified in this study were among the commonest *KRAS* mutations, in agreement with other studies [17, 28, 33]. Our data showed that no significant association between *KRAS* mutations and age, gender, histologic grade or tumor site. However, some previous reports found that the rate of *KRAS* mutations were higher in females than males [10, 42] which was opposite to the study of Poehlmann et al [17].

Our data showed a superior consistency between MAS-PCR assay and pyrosequencing (k = 0.947). Seven discordances were found according to pyrosequencing results. Direct sequencing and pyrosequencing revealed the same mutant alleles, which comprised of 3 cases of G12D, 3 cases of G12A, and 1 case of G12R. One possible outcome is the formation of cross-dimers between primers leading to biased amplification, which may reduce sensitivity of the MAS-PCR assay [43]. In addition, FFPE tissue samples may have degraded nucleic acids due to the fixation process, and the effect of cross-linking fixatives on the nucleic acids is detrimental causing PCR inhibition [44, 45]. Interestingly, our MAS-PCR assay showed high analytical sensitivity of detection, with approximately 1–2% mutant allele detected based on DNA mixing experiments using genomic DNA isolated from plasmid cloned DNA. However, such sensitivity might be affected considering the use of clinical samples that are usually complicated with the presence of PCR interfering components in the samples. Nevertheless, most interfering components could be eliminated by DNA purification using FFPE tissue kits as we employed in this study and as previously described [46, 47]. Moreover, the MAS-PCR assay that we developed has a higher sensitivity than that reported for direct sequencing and HRM (i.e., 5% to 20%), and is equivalent to that reported for pyrosequencing and commercial molecular kits (i.e., 1% to 5%) [14, 18, 38]. Furthermore, we found a good precision and reproducibility of the developed assay, as four repeated run yielded the same results. In addition, MAS-PCR is a rapid assay requiring only < 4 h to complete the assay (excluding DNA isolation); it is inexpensive, costing approximately $8 per test. Moreover, the assay employs only a PCR instrument, and does not require a high level of technical expertise and specialized equipment.

## Conclusions

In conclusion, we developed a MAS-PCR assay for detection of the seven most common mutations in codons 12 and 13 of the *KRAS* gene. MAS-PCR assay is a DNA-based protocol that was easy to perform, being rapid, cost-effective, highly sensitive and highly specific. An assay with these characteristics is important for analysis of clinical samples, such as FFPE tissues, in particular to assist clinicians in predicting the clinical course of monoclonal anti-EGFR antibody treatment of mCRC patients.
